# Timescales of learning in the basal ganglia and the hippocampus

**DOI:** 10.3389/fnbeh.2013.00098

**Published:** 2013-08-01

**Authors:** Daniele Ortu, Ida M. Skavhaug, Manish Vaidya

**Affiliations:** ^1^Neurobehavioral Laboratory, Department of Behavior Analysis, University of North TexasDenton, TX, USA; ^2^Robotics, Autonomous Systems, and Controls Laboratory, University of California DavisDavis, CA, USA

Historically there has been a distinction between basal ganglia dependent and hippocampus dependent memory systems (e.g., Squire, 1992). It is commonly understood that whilst the striatum—located in the basal ganglia—supports learning of stimulus-response associations through environmental feedback, the hippocampus supports a distinct long-term episodic memory system. Recent theoretical and experimental considerations have, however, led to the hypothesis that hippocampal function is, similarly to the striatum, dependent on signals from subcortical nuclei that are sensitive to environmental forms of feedback such as reinforcing stimulation (e.g., Gaffan, 2002; Martig and Mizumori, 2011). Compelling evidence of the role of reinforcing stimulation on hippocampal mediated learning also comes from experiments analyzing the theta rhythm (5–12 Hz) in the hippocampus of the behaving rat (for a review see Vertes, 2005). When the rat explores an environment theta oscillations lead to a weak (protein synthesis independent) form of long term potentiation (LTP), determining relatively short (1–3 h) changes in the involved synaptic efficacies. If, however, theta oscillations during exploratory behavior are paired with presentation of reinforcers, the modulatory pathways converging to the hippocampus (Gasbarri et al., 1994; Lisman and Grace, 2005; Martig and Mizumori, 2011) allow long term retention of learning. In other words, learning is facilitated on one level by active responding (e.g., locomotion)—correlated with theta rhythm—and is further enhanced by presentation of reinforcing stimulation which triggers neuromodulation of hippocampal activity. Importantly, recent research carried out on human participants using intracranial EEG (Lega et al., 2012) suggests that the human theta rhythm measured during learning may be equivalent to the theta oscillations described in rats during spatial learning.

A newly published study by Foerde et al. (2013) also supports the idea that environmental feedback plays an important role with respect to both basal ganglia and hippocampus based learning. Specifically Foerde et al. examined behavioral differences across clinical groups whose pathologies were known to selectively impair the neural structures of interest. They compared the behavioral performance of participants affected by hippocampal lesions, due to anoxia or herpes encephalitis, to the performance of patients affected by Parkinson's, which damage involves the striatum. Foerde et al. clearly demonstrated that both the striatum and the hippocampus are sensitive to feedback learning—although at a different timescale. Parkinsonian patients, with basal ganglia damage, showed preserved feedback learning when the response-feedback interval was long (7 s) but not when the response-feedback interval was short (1 s). Conversely patients with hippocampal damage were impaired at feedback learning at short but not long intervals.

The failure to learn from delayed feedback in patients with hippocampal lesions could be related to the loss of function of area CA1, since CA1 receives afferents from areas known to respond phasically to reinforcing stimulation (Gasbarri et al., 1994; Martig and Mizumori, 2011) and appears to be critically involved in temporal analysis when there are long delays among relevant stimuli (e.g., Hunsaker and Kesner, 2008; Farovik et al., 2010). Because CA1 is involved in temporal analysis in presence of long delays and receives a learning signal from areas that respond phasically to reinforcers such as the ventral tegmental area (VTA), it might serve a function in keeping the reinforcer effective during the 7 s delay in the experiment by Foerde et al. (2013). For patients affected by Parkinson's, however, the ability to learn from delayed feedback might be preserved, as area CA1 within the hippocampus is still functional and receiving “environmental” feedback from the VTA, while fast learning mediated by the basal ganglia may be impaired because of the striatal damage caused by the dopaminergic depletion.

Overall, the results described by Foerde et al. (2013) provide evidence that both the hippocampus and the striatum are involved in reinforcement learning. The sensitivity to feedback at different timescales, provided by the striatum and the hippocampus, guarantees the ability to behave adaptively within natural settings. Non-experimental environments are in fact necessarily characterized by variability in the latency of consequences to individual behavior. Accordingly, different neural structures have been shown to be sensitive to different timescales (for a review, see Buhusi and Meck, 2005). The sensitivity to different timescales may be an interesting case of degeneracy (e.g., Edelman and Gally, 2001), in which different neural structures share similar, and perhaps overlapping, functions. From this perspective a significant question that remains to be answered, as Foerde et al. point out, regards the inability of the hippocampus to mediate reinforcement based learning with short delays. Is this inability due to an intrinsic sensitivity of the hippocampus to increasing delays (Picchioni et al., 2007), or is hippocampus-mediated reinforcement at short delays inhibited by prefrontal cortex mechanisms? Prefrontal areas have been in fact found to be involved in inhibitory functions that might regulate a “competition” between learning systems (Poldrack and Rodriguez, 2004). A more precise measurement of the two structures' sensitivities to delay would help answering such a question. In future research, the duration of the response-feedback interval might therefore be manipulated parametrically (e.g., from 1 to 10 s at 1 s intervals), to assess if the timescale in which the striatum and the hippocampus operate are entirely distinct (or if there is a degree of overlap in sensitivity to feedback delay). It seems, however, that at least some of the operations carried out by the hippocampus could not be carried out by the striatum. Degeneracy across the hippocampus and the striatum due to common sensitivity to feedback learning may therefore only be partial as confirmed by other neuropsychological studies emphasizing important differences between the two learning systems (e.g., Myers et al., 2003). In spite of these clear functional differences between the two learning systems, the general picture that emerges from Foerde et al. and the related literature suggests that the basal ganglia and the hippocampus share overlapping sensitivity to reinforcement signals, although at a different timescale.
